# Urinary Tract Infections and Bacterial Multidrug Resistance in Kidney Transplant Impact on Function and Graft Survival

**DOI:** 10.3390/clinpract15110215

**Published:** 2025-11-19

**Authors:** Hernán Javier Pájaro Huertas, María Viviana Pantoja Echeverri, Gustavo Aroca Martínez, Carlos Guido Musso, Alex Dominguez Vargaz, Henry J. González-Torres

**Affiliations:** 1 Centro de Investigaciones en Ciencias de la Vida, Facultad de Ciencias de la Salud, Universidad Simón Bolívar, Barranquilla 080001, Colombia; hernan.pajaro@unisimon.edu.co (H.J.P.H.); maria.pantojae@unisimon.edu.co (M.V.P.E.); gustavo.aroca@unisimon.edu.co (G.A.M.); carlos.musso@hospitalitaliano.org.ar (C.G.M.); 2 Departamento de Nefrología, Clínica de la Costa, Barranquilla 080001, Colombia; 3 Nephrology Department, Hospital Italiano de Buenos Aires, Buenos Aires C1000, Argentina; 4 Data Analysis and Mining Department, Data & Project Consulting Service, Barranquilla 080001, Colombia; aadominguez17@hotmail.com

**Keywords:** kidney transplantation, urinary tract infection, multidrug resistance, renal function, graft survival

## Abstract

**Objective**: This study aimed to evaluate the sociodemographic, clinical, paraclinical, and microbiological characteristics of kidney transplant recipients with and without urinary tract infection (UTI) and determine their impact on renal function and graft survival in a referral center in Atlántico, Colombia. **Methods**: We conducted a retrospective, observational, analytical study including 163 kidney transplant recipients between 2015 and 2020. Clinical and microbiological variables were compared according to UTI status. Renal function was assessed using estimated glomerular filtration rate (eGFR). Graft survival was analyzed with Kaplan–Meier curves, and predictors of graft loss were identified using Cox regression models. **Results**: UTI prevalence was 17.8% (29/163), with a higher proportion of women in the UTI (+) group compared to the UTI (−) group (62% vs. 34%, *p* = 0.004). *Escherichia coli* (59%) and *Klebsiella* spp. (31%) were the predominant pathogens, with MDR in 66% of isolates and carbapenem resistance in 28%. Patients with UTIs had significantly lower baseline and follow-up eGFR (*p* ≤ 0.002), yet five-year graft survival was comparable (93% vs. 91%, *p* = 0.54). Baseline eGFR (HR: 0.95, *p* < 0.001) and institutional referral (HR: 9.7, *p* = 0.010) were independent predictors of graft loss, whereas UTIs were not associated with increased risk. **Conclusions**: Post-transplant UTIs in Atlántico were characterized by high antimicrobial resistance and reduced renal function, but did not affect graft survival. Antimicrobial stewardship and institutional optimization strategies are essential to improve outcomes in this vulnerable population.

## 1. Introduction

Kidney transplantation is the treatment of choice for end-stage chronic kidney disease, as it provides significant advantages in survival and quality of life compared with dialysis therapy [1]. However, the success of this procedure is limited by infectious complications, which remain among the leading causes of morbidity and mortality in transplant recipients [2]. Among these, urinary tract infections (UTIs) are the most frequent, with a reported incidence of up to 40% during the first year post-transplant [3,4].

The high frequency of UTIs in this setting can be explained by factors such as pharmacological immunosuppression, perioperative urological manipulation, bladder catheterization, and anatomical alterations of the graft itself [5]. These infections not only lead to recurrent hospitalizations and impaired quality of life, but have also been associated with acute rejection, graft loss, and an increased risk of mortality [6].

A particularly concerning issue is the growing prevalence of multidrug-resistant (MDR) uropathogens, especially extended-spectrum beta-lactamase (ESBL)-producing *Escherichia coli* and *Klebsiella* spp., as well as carbapenem-resistant strains [7,8]. Recent studies have shown that the proportion of multidrug-resistant (MDR) bacteria in post-transplant UTIs exceeds 50%, complicating clinical management and limiting available therapeutic options [9]. Moreover, antimicrobial resistance has been linked to prolonged hospital stays, higher healthcare costs, and an increased risk of systemic complications [10].

Nevertheless, evidence regarding the true impact of UTIs and antimicrobial resistance on renal function and graft survival remains inconsistent. Some studies have demonstrated an association between recurrent UTIs and an increased risk of long-term graft loss [11], whereas others have found no significant differences in survival, suggesting that the effect may be more closely related to baseline renal function and the clinical condition of the recipient [12]. This heterogeneity in findings reflects differences in study design, follow-up periods, and epidemiological contexts, particularly in middle-income countries, where bacterial resistance patterns are more aggressive and access to last-line antimicrobials is limited [13].

In Latin America, data on post-transplant UTIs and resistance profiles are scarce. In Colombia, and particularly in the Atlántico region, the burden of antimicrobial resistance in urinary tract infections in the general population is high, raising questions about its specific impact on immunocompromised patients such as kidney transplant recipients [14]. In this context, it is critical to characterize the clinical and microbiological profiles of UTIs in kidney transplant recipients and to assess their impact on renal function and graft survival.

The present study aimed to evaluate the sociodemographic, clinical, and microbiological characteristics of kidney transplant patients in Atlántico, with and without a diagnosis of UTI, determining the prevalence of multidrug-resistant microorganisms and their relationship with functional outcomes and graft survival. This work seeks to provide local evidence to inform antimicrobial surveillance programs and to optimize therapeutic strategies in a highly vulnerable population.

## 2. Materials and Methods

### 2.1. Study Design

We conducted an observational, retrospective, and analytical study in kidney transplant recipients at a referral center in the Atlántico Department, Colombia. The primary objective was to evaluate the relationship between UTIs, associated microbiological profiles, and clinical outcomes, with particular emphasis on renal function and graft survival.

### 2.2. Study Population

The cohort included adult patients enrolled in the kidney transplant program who had a minimum follow-up duration of five years. All kidney transplant recipients who underwent transplantation between January 2015 and December 2020 and had complete clinical records in the institutional database were eligible for inclusion. Exclusion criteria were: loss to follow-up within the first 30 days post-transplant, absence of microbiological data, or incomplete electronic health records. A total of 163 patients were included in the final analysis, of whom 29 had a confirmed diagnosis of post-transplant UTI.

### 2.3. Definitions and Variables

UTI was defined as the presence of compatible clinical signs or symptoms such as fever (>38 °C), dysuria, urinary urgency or frequency, suprapubic or flank pain, or systemic manifestations of infection (e.g., chills, malaise) accompanied by microbiological isolation of a uropathogen (≥10^4^ CFU/mL) in urine or bladder catheter specimens. Asymptomatic bacteriuria was not classified as UTI and was excluded from the analysis, in accordance with current Infectious Diseases Society of America (IDSA) [15] and American Society of Transplantation (AST) guidelines [16]. UTIs were further classified as community-acquired (CAI) or healthcare-associated (HAI) based on the Centers for Disease Control and Prevention (CDC) criteria

The following variables were collected: sociodemographic (age, sex, place of residence), clinical (comorbidities, donor type, ischemia time, HLA compatibility), laboratory (baseline and follow-up estimated glomerular filtration rate [eGFR]), microbiological (isolated microorganisms and resistance profiles), and clinical outcomes (graft loss and mortality).

### 2.4. Immunosuppression Regimens

All patients received perioperative induction therapy followed by triple-drug maintenance immunosuppression. Induction consisted of basiliximab (20 mg intravenously on day 0 and day 4) in low-to-moderate immunologic risk recipients, and rabbit anti-thymocyte globulin (1.5 mg/kg/day for 4 days) in high-risk cases (e.g., repeat transplants, high PRA, or African ancestry). Maintenance therapy included tacrolimus (target trough levels: 8–10 ng/mL in months 0–3, 5–8 ng/mL thereafter), mycophenolate mofetil (1–1.5 g twice daily), and prednisone (starting at 20 mg/day, tapered to 5 mg/day by 6 months) [17]. Steroid avoidance or CNI minimization protocols were not used during the study period.

### 2.5. Data Collection

Information was extracted from institutional electronic health records, transplant program registries, and microbiology laboratory reports. Diagnoses of UTIs and antimicrobial susceptibility profiles were validated by infectious disease specialists to ensure data accuracy.

### 2.6. Microbiological Analysis

Microorganisms were identified using standardized automated laboratory systems (Phoenix™ M50 (BD Diagnostics, Sparks, MD, USA)). Antimicrobial susceptibility testing was performed by disk diffusion and/or broth microdilution in accordance with Clinical and Laboratory Standards Institute (CLSI) guidelines [18]. Minimum inhibitory concentrations (MICs) were determined for relevant antimicrobial agents when indicated by the testing platform. Phenotypic confirmation of ESBL production was performed using the CLSI-recommended combination disk method, with cefotaxime, ceftazidime, and cefepime tested both alone and in combination with clavulanic acid [18].

Isolates were classified as MDR, extensively drug-resistant (XDR), or carbapenem-resistant (CR) according to the international consensus criteria established by Magiorakos et al. [19]. MDR was defined as non-susceptibility to ≥1 agent in ≥3 antimicrobial classes; XDR as non-susceptibility to ≥1 agent in all but ≤2 classes (i.e., isolates remained susceptible to agents in only one or two classes); and CR as non-susceptibility to ≥1 carbapenem agent (e.g., imipenem or meropenem) [19]. All interpretations were based on CLSI clinical breakpoints in effect at the time of testing.

### 2.7. Assessment of Renal Function and Graft Survival

Renal function was assessed by estimating glomerular filtration rate (eGFR) using the CKD-EPI equation at four pre-specified time points: baseline (pre-UTI) and three follow-up visits within the five-year post-transplant period. Only patients with eGFR measurements available at all four time points were included in the longitudinal analysis. Graft survival was defined as the time from transplantation to documented graft loss or end of follow-up, whichever occurred first.

### 2.8. Statistical Analysis

Normality of continuous variables was assessed using the Kolmogorov–Smirnov test. Continuous data are presented as medians with interquartile ranges (IQRs), and categorical variables as frequencies (percentages). Between-group comparisons (UTI[+] vs. UTI[–]) were performed using the Wilcoxon rank-sum test for continuous variables and chi-square or Fisher’s exact tests, as appropriate, for categorical variables. Longitudinal changes in eGFR were evaluated with the Friedman test. Kidney graft survival was estimated using the Kaplan–Meier method and compared between groups with the log-rank test. Predictors of kidney graft loss were identified using Cox proportional hazards regression, with results expressed as hazard ratios (HRs) and 95% confidence intervals (CIs). The proportional hazards assumption was verified using Schoenfeld residuals and log-log survival plots, with no significant violations observed. Given the limited number of graft loss events (n = 12), the risk of model overfitting was considered; thus, non-significant associations are interpreted as hypothesis-generating rather than conclusive. A two-sided *p*-value < 0.05 was considered statistically significant. All analyses were performed using R statistical software (version 4.4.0; R Foundation for Statistical Computing, Vienna, Austria) [20].

### 2.9. Ethical Considerations

The study was conducted in accordance with the ethical principles of the Declaration of Helsinki and Colombian national regulations for health research (Resolution 8430 of 1993, Ministry of Health). All data were anonymized to ensure participant confidentiality, and researchers had no access to personal identifiers. Given the retrospective, non-interventional design, the requirement for informed consent was waived. The study protocol was reviewed and approved by the Scientific Committee of the Faculty of Health Sciences, Universidad Simón Bolívar, and by the Institutional Research Ethics Committee (IRB) of the participating clinic.

## 3. Results

### 3.1. Baseline Characteristics

A total of 163 kidney transplant recipients were included, of whom 29 (17.8%) developed a UTI. Baseline sociodemographic and clinical characteristics are summarized in Table 1. The majority of patients were male (61%) and referred from external institutions (60%), with a median age of 44 years (range: 18–75). Age did not differ significantly between UTI (+) and UTI (−) groups (40 vs. 44 years, *p* = 0.32). However, women were significantly overrepresented in the UTI (+) group (62% vs. 34%, *p* = 0.004). Institutional referral was also more common among UTI (+) patients (62% vs. 36%, *p* = 0.009).

**Figure 1:**
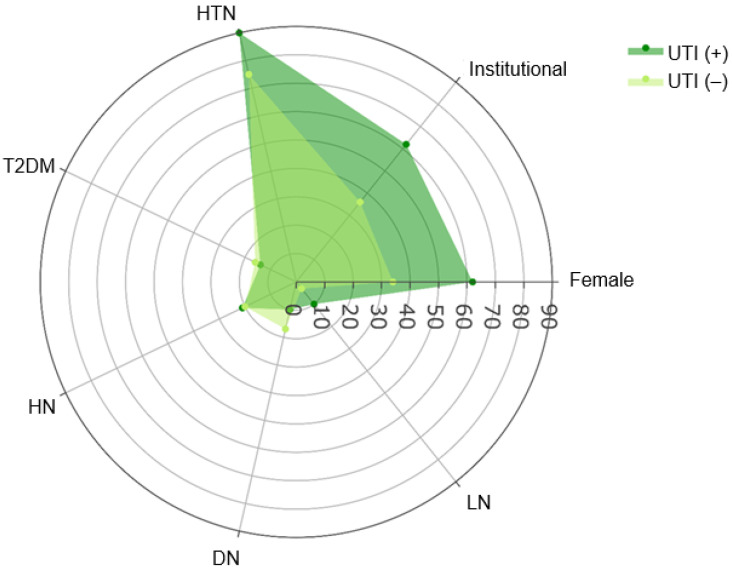
Distribution of demographic and clinical parameters in kidney transplant recipients according to UTI diagnosis. UTI: Urinary tract infection.

Hypertension was the most prevalent comorbidity overall (78%), with a higher but non-significant frequency in the UTI (+) group (90% vs. 75%, *p* = 0.09) (Figure 1). The distribution of primary kidney diseases including hypertensive nephroangiosclerosis, diabetic nephropathy, and lupus nephritis did not differ significantly between groups (all *p* > 0.05).

### 3.2. Baseline Renal Function Pathological Characteristics and Outcomes

**Figure 2:**
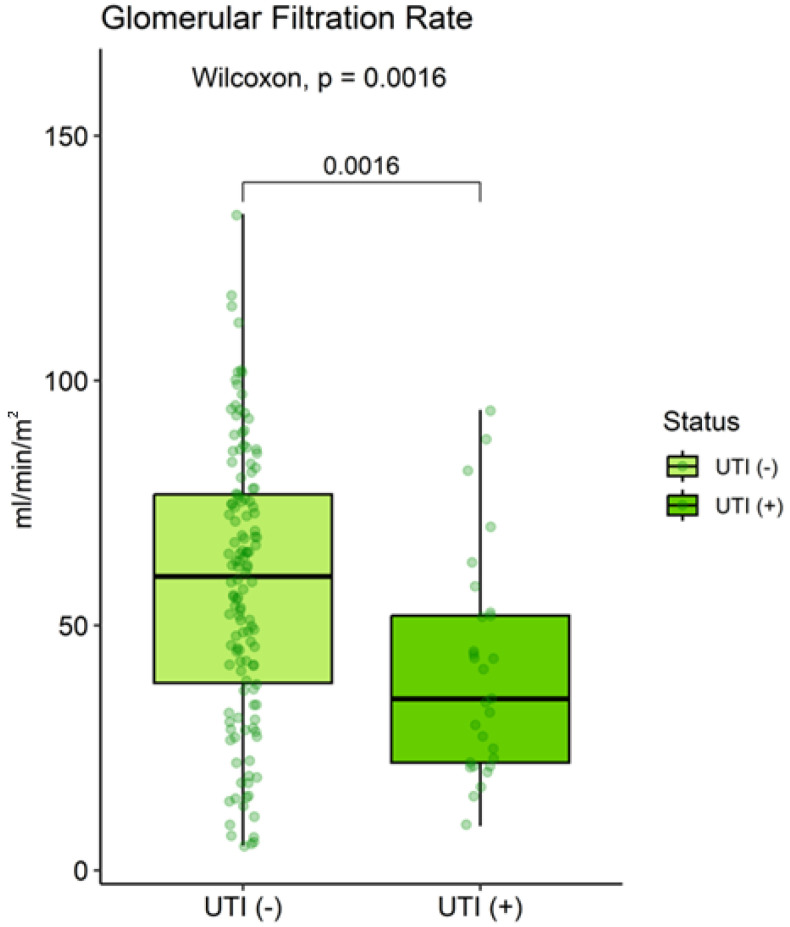
Comparison of baseline estimated glomerular filtration rate in kidney transplant recipients according to UTI diagnosis. UTI: Urinary tract infection.

Baseline eGFR was significantly lower in the UTI (+) group (median: 35.0 vs. 60.0 mL/min/1.73 m^2^, *p* = 0.002) (Figure 2). No significant differences were observed in donor type (deceased: 55% vs. 49%, *p* = 0.53), ischemia time (8.7 vs. 9.9 h, *p* = 0.62), panel-reactive antibodies (*p* = 0.64), or HLA matching (all *p* > 0.4) (Table 2). Graft loss occurred in 12 patients (7.4%). Acute rejection episodes, diagnosed by protocol or indication biopsy according to Banff criteria, were observed in 14 patients (8.6%) during follow-up, with no significant difference between UTI (+) and UTI (−) groups (10.3% vs. 8.2%; *p* = 0.72).

**Figure 3:**
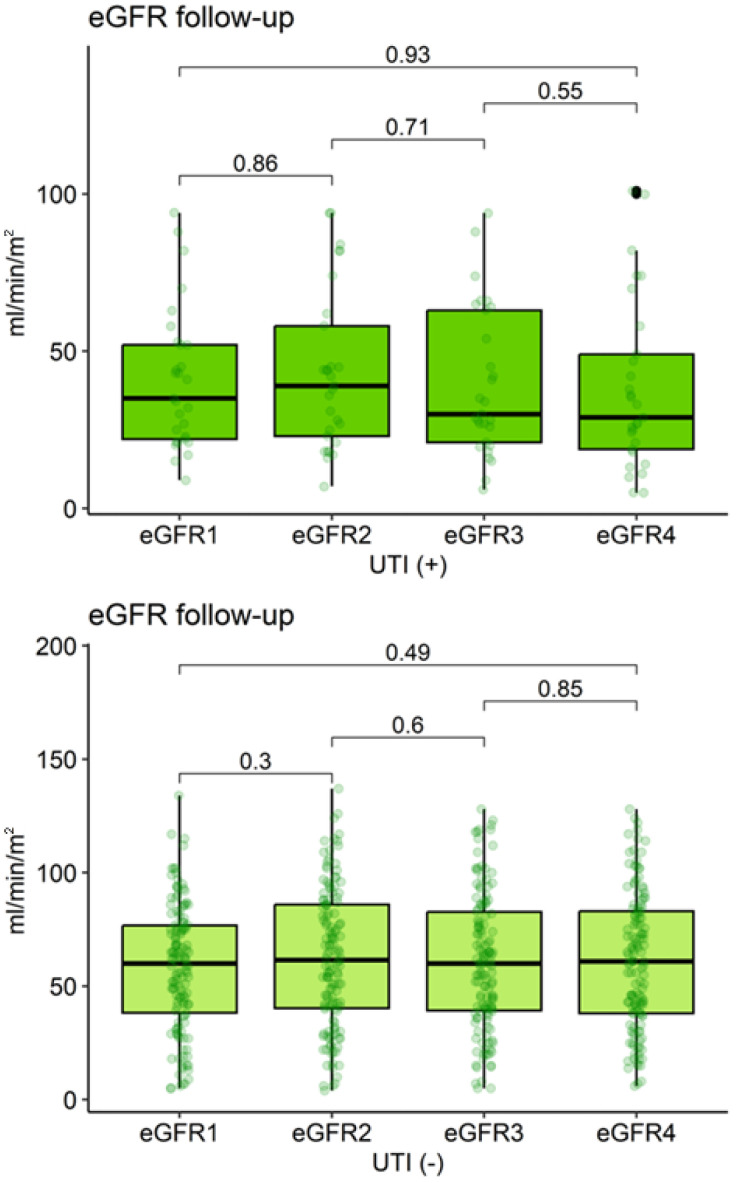
Evolution of estimated glomerular filtration rate (eGFR) in kidney transplant recipients according to urinary tract infection (UTI) diagnosis. eGFR: Estimated glomerular filtration rate; UTI: Urinary tract infection. The black dots indicate outliers.

During follow-up, eGFR remained consistently lower in the UTI (+) group at all time points (eGFR_2_: *p* = 0.002; eGFR_3_ and eGFR_4_: *p* < 0.001) (Table 3, Figure 3). However, eGFR did not change significantly over time within either group (UTI (−): *p* = 0.59; UTI (+): *p* = 0.68).

### 3.3. Microbiological Profile of UTIs

Table 4 compares the clinical characteristics and microbial profiles of kidney transplant recipients with UTI (n = 29) stratified by sex. Hospitalization was more frequent in men (18% vs. 5.6%, *p* = 0.5), though not statistically significant. Infection source (urine vs. catheter) and acquisition type (healthcare-associated vs. community-acquired) were similar between sexes (all *p* > 0.9).

**Figure 4:**
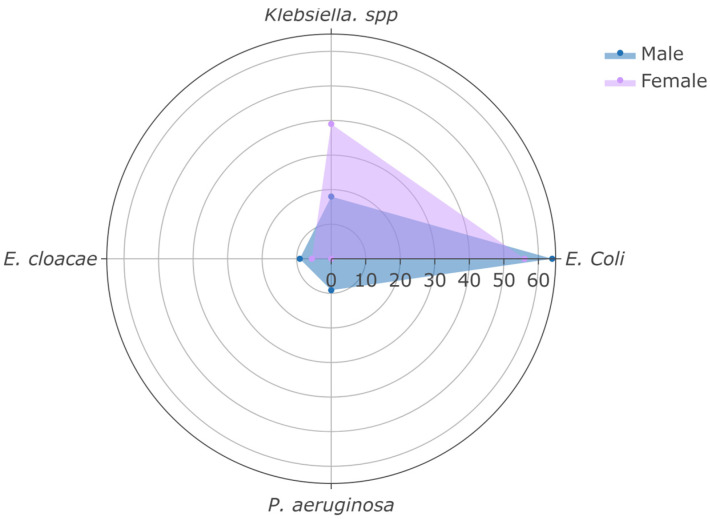
Distribution of the most frequently isolated microorganisms in kidney transplant recipients with urinary tract infection (UTI), stratified by sex.

All isolates were Gram-negative. *E. coli* was the predominant pathogen (59%), followed by *Klebsiella* spp. (31%), with no significant sex-based differences in pathogen distribution (Figure 4).

**Figure 5:**
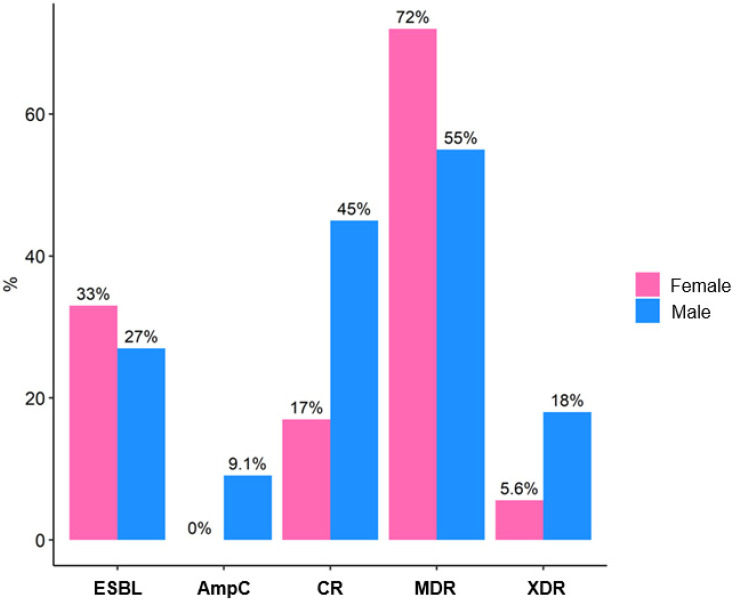
Microbial resistance profile in kidney transplant recipients with urinary tract infection, stratified by sex. ESBL: Extended-spectrum beta-lactamases; CR: Carbapenem resistance; MDR: Multidrug-resistant; XDR: Extensively drug-resistant; AmpC: AmpC-type beta-lactamases.

Susceptibility to individual antibiotics is detailed in Table 5. Amikacin retained the highest activity (83% susceptible), whereas fluoroquinolones showed limited efficacy (<30% susceptibility). Antimicrobial resistance was prevalent: 66% of isolates were MDR, and 28% exhibited CR. Although men showed higher rates of CR (45% vs. 17%) and XDR strains (18% vs. 5.6%), these differences were not statistically significant (all *p* > 0.2) (Table 5, Figure 5).

### 3.4. Graft Survival

**Figure 6:**
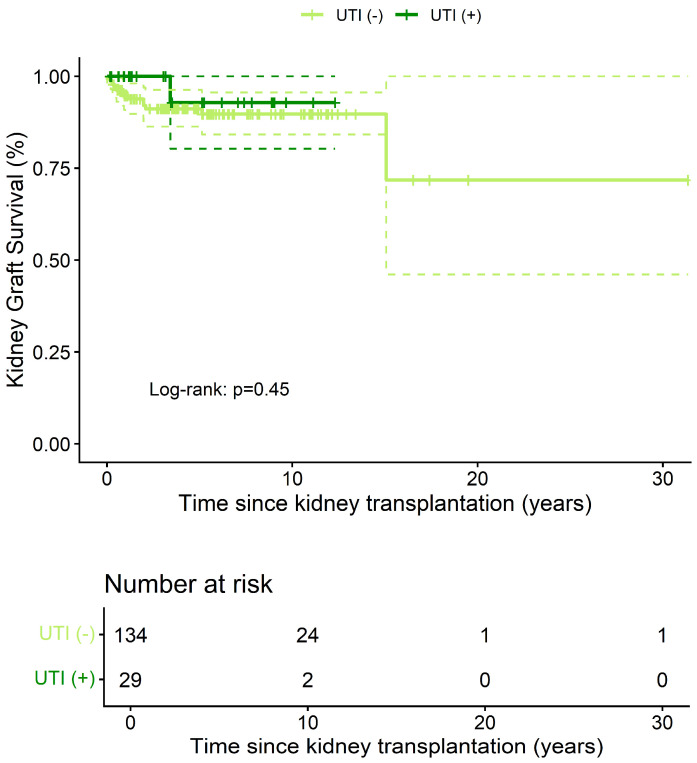
Comparison of kidney graft survival in transplant recipients according to UTI diagnosis. Kaplan–Meier analysis. Solid lines indicate the Kaplan–Meier graft survival estimates; dotted lines represent the 95% confidence intervals. UTI: Urinary tract infection.

Figure 6 shows a comparison of kidney graft survival in transplant recipients, stratified by UTI diagnosis, using Kaplan–Meier curves. This survival analysis assessed the probability of kidney graft survival over time (in years) post-transplant, comparing UTI (+) and UTI (−) groups. The log-rank test revealed no statistically significant differences in kidney graft survival between groups (*p* = 0.45).

At 5 years post-transplant, the probability of graft survival was 91% (95% CI: 86–96%) in the UTI (−) group (number at risk = 62, graft losses = 11) and 93% (95% CI: 80–100%) in the UTI (+) group (number at risk = 12, graft losses = 1). Among the 12 graft losses, causes included chronic antibody-mediated rejection (n = 5), recurrent glomerular disease (n = 3; including focal segmental glomerulosclerosis and membranous nephropathy), acute T-cell–mediated rejection (n = 2), thrombotic microangiopathy (n = 1), and unknown etiology (n = 1). No losses were attributed to UTI or urosepsis

### 3.5. Risk Factors for Graft Loss

In a Cox regression analysis to identify risk factors associated with graft loss in 163 kidney transplant recipients, multiple demographic, clinical, and pathological characteristics were evaluated (Table 6). Baseline eGFR was a significant predictor of graft loss (HR: 0.95, 95% CI: 0.92–0.97, *p* < 0.001), indicating that each 1 mL/min/m^2^ increase in baseline eGFR reduced the risk by 5%. Institutional referral was also significantly associated with a higher risk of graft loss (HR: 9.7, 95% CI: 2.03–17, *p* = 0.01). Other factors, including age (HR: 0.93, *p* = 0.4), male sex (HR: 0.65, *p* = 0.6), hypertension (HR: 0.18, *p* = 0.12), living donor (HR: 1.28, *p* = 0.8), presence of UTI (HR: 0.11, *p* = 0.2), and primary kidney diseases (all *p* > 0.05), were not significantly associated with graft loss.

## 4. Discussion

In this retrospective cohort study of 163 kidney transplant recipients in the Atlántico region of Colombia, we observed a UTI prevalence of 17.8%, with a marked predominance among women and patients referred from external institutions. eGFR was significantly lower in patients who developed UTI and remained reduced during follow-up, although this did not translate into worse graft survival at five years. *Escherichia coli* and *Klebsiella* spp. were the predominant uropathogens, with a high frequency of MDR (66%), including CR and the presence of XDR strains. In multivariate analysis, lower baseline eGFR and institutional referral status were significant independent predictors of graft loss, whereas UTI itself was not associated with an increased risk.

The UTI prevalence in our cohort (17.8%) was lower than that reported in European and North American series, where cumulative incidence typically ranges from 20% to 40% within the first post-transplant year [2,3]. Reported rates vary widely across studies: a multicenter study described a first-year prevalence of approximately 25% [13], while reports from Saudi Arabia noted rates exceeding 30% [9]. These differences may reflect differences in antimicrobial prophylaxis protocols, access to microbiological diagnostics, and case definitions for UTI.

The overrepresentation of women in the UTI group aligns with global epidemiological patterns, attributable to the anatomical and physiological susceptibility of the female urinary tract [4,21]. Hypertension, the most common comorbidity (78%), reflects the high cardiovascular burden in the transplant population and has been consistently reported as a risk factor for post-transplant complications [1]. The finding of significantly lower baseline eGFR among patients with UTI aligns with prior studies suggesting that impaired renal function predisposes individuals both to infection and to a poorer functional prognosis [6,22]. However, our results show that UTI per se did not increase graft loss, consistent with a Spanish clinical trial that found no differences in graft survival when asymptomatic bacteriuria was systematically treated [11].

Regarding microbiological profiles, the predominance of *E. coli* (59%) and *Klebsiella* spp. (31%) is consistent with the prior literature [8,12]. Nevertheless, the high prevalence of MDR (66%) in our cohort exceeds that reported in European series (30–40%) [23] and more closely resembles patterns observed in middle-income countries, where high antimicrobial pressure and deficiencies in infection control practices promote the emergence and spread of resistant pathogens [7,10]. Particularly concerning is the prevalence of CR (28%), which markedly surpasses rates reported in North American cohorts, where CR remains below 10% [24].

In terms of outcomes, five-year graft survival was high and comparable between patients with and without UTI (93% vs. 91%). This finding contrasts with studies suggesting that recurrent UTIs negatively impact renal function and graft survival [25], but is in line with recent research highlighting baseline eGFR and other clinical factors as stronger predictors of outcomes [26].

Our results suggest that, although UTIs are frequent and associated with short- and mid-term eGFR decline, they do not necessarily compromise long-term graft survival when diagnosed and treated promptly. Possible explanations include early infection detection, the availability of active antimicrobials against resistant uropathogens, and strict follow-up in specialized transplant programs [27]. The significant association between institutional referral and graft loss (HR: 9.7) raises hypotheses related to the quality-of-care processes, differences in postoperative follow-up, and adherence to immunosuppressive protocols. In our setting, “institutional referral” denotes patients transplanted at our center, but originally managed elsewhere prior to transplantation. This group may be subject to referral bias, wherein external centers transfer higher-risk or more complex cases, potentially confounding the observed association.

Moreover, fragmented pre-transplant care may lead to suboptimal preparation, delayed immunosuppression initiation, or weaker patient–provider relationships, all of which can compromise long-term adherence and outcomes [28,29]. Although we adjusted for key clinical variables, unmeasured confounders such as socioeconomic status, access to medications, transportation barriers, or social support are likely unevenly distributed between referral groups and may partially explain this finding. Notably, studies in transplant systems with integrated care pathways report better outcomes, underscoring the importance of continuity and coordination in post-transplant management [28]. Future prospective studies should incorporate granular data on care trajectories and social determinants to disentangle true causal effects from referral-related confounding.

Antimicrobial susceptibility profile in our cohort highlights significant therapeutic constraints. Amikacin retained the highest activity (83% susceptible), fluoroquinolone susceptibility was alarmingly low (24–28%), and cephalosporin susceptibility ranged from 34% to 41%, likely due to the high prevalence of ESBL producers (31%), a pattern consistent with other Latin American reports [30,31]. Piperacillin-tazobactam showed moderate activity (52%), suggesting a potential role in empirical therapy where local resistance permits. These patterns highlight the critical need for center-specific antibiograms to guide empirical treatment in kidney transplant recipients, as reliance on historical or regional assumptions may lead to inadequate initial therapy, known as a known risk factor for poor outcomes in immunocompromised hosts [27]

The detection of XDR isolates in 10% of cases should be interpreted as a warning signal. Evidence indicates that XDR bacterial infections in transplant recipients are associated with increased mortality and severely limited treatment options [32]. Although no attributable deaths occurred in our cohort, this resistance phenotype represents a serious public health threat that warrants rigorous antimicrobial surveillance and stewardship.

This study has several strengths: (i) comprehensive integration of sociodemographic, clinical, laboratory, and microbiological data; (ii) longitudinal follow-up of up to five years, enabling assessment of hard endpoints such as graft loss; and (iii) detailed characterization of antimicrobial resistance patterns in a region with limited published data.

Nonetheless, several limitations must be acknowledged. First, the retrospective design precludes causal inference and is susceptible to information bias related to the completeness and accuracy of electronic health records. Second, the number of patients with UTI (n = 29) was relatively small, limiting statistical power to detect associations with rare outcomes such as graft loss or mortality. Third, we did not assess UTI recurrence or adherence to antimicrobial therapy, both of which are known to influence the development of multidrug resistance, renal function decline, and long-term graft outcomes. In our cohort, patients were classified based on the occurrence of at least one episode of symptomatic UTI during the five-year follow-up period; however, due to inconsistent documentation of repeat episodes in clinical and microbiological records, we were unable to perform a granular analysis of recurrence frequency, timing, or treatment response. Future prospective studies should systematically capture these variables to better elucidate their impact on transplant outcomes. Furthermore, the low number of graft loss events (n = 12) limits the precision of our Cox regression model, particularly for less common exposures such as UTI (+). The resulting wide confidence intervals preclude definitive conclusions about effect size, but are consistent with the absence of a clinically meaningful increase in risk.

The findings have important practical implications. The high prevalence of MDR and carbapenem resistance mandates revision of prophylaxis and empirical treatment regimens in kidney transplant recipients, emphasizing rational antimicrobial use and adherence to local susceptibility guidelines. The observation that UTI did not increase graft loss suggests that priority should focus on optimizing baseline renal function and standardizing institutional follow-up protocols.

From a public health perspective, our results reinforce the need for epidemiological surveillance programs in transplant-related infections and for regional collaborative networks to share antimicrobial resistance data, as already implemented in Europe and North America [14]. Future multicenter and prospective studies in Colombia and Latin America should explore UTI recurrence, the impact of XDR bacterial infections on mortality, and the effectiveness of infection control interventions in transplant programs [23,31]. Additionally, analysis of inflammatory and tubular dysfunction biomarkers as early predictors of renal damage would be valuable in this context.

## 5. Conclusions

In this cohort of kidney transplant recipients in the Atlántico region of Colombia, UTIs occurred in 17.8% of patients and were predominantly caused by *Escherichia coli* and *Klebsiella* spp., with a high prevalence of MDR isolates. Patients with UTI exhibited significantly lower eGFR at baseline and throughout follow-up; however, this functional decline did not translate into reduced five-year graft survival compared with non-infected recipients. Baseline renal function and institutional referral status emerged as the strongest independent predictors of graft loss, whereas UTI was not associated with an increased risk of adverse outcomes. These findings suggest that, beyond the acute effects of infection, clinical efforts should prioritize optimizing early renal function and standardizing post-transplant care pathways, particularly for patients referred from external centers. From both clinical and public health perspectives, the high burden of antimicrobial resistance underscores the urgent need to strengthen microbiological surveillance, implement antimicrobial stewardship policies, and enhance infection prevention strategies in transplant populations. Future multicenter, prospective studies are essential to validate these observations and to evaluate targeted interventions aimed at mitigating the impact of resistant bacterial infections in kidney transplantation.

## Figures and Tables

**Table 1:** Sociodemographic and clinical characteristics of kidney transplant recipients according to UTI diagnosis.

Characteristic	Overall n = 163 ^1^	UTI (−) n = 134 ^1^	UTI (+) n = 29 ^1^	*p*-Value
Age (years)	44 (18–75)	44 (30–56)	40 (18–75)	0.3 ^2^
Sex				0.004 ^3^
Female	63 (39%)	45 (34%)	18 (62%)	
Male	100 (61%)	89 (66%)	11 (38%)	
Referral				0.009 ^3^
External	97 (60%)	86 (64%)	11 (38%)	
Institutional	66 (40%)	48 (36%)	18 (62%)	
Comorbidities				
Diabetes				>0.9 ^4^
DM1	3 (1.8%)	3 (2.2%)	0 (0%)	
DM2	26 (16%)	22 (16%)	4 (14%)	
Hypertension	127 (78%)	101 (75%)	26 (90%)	0.093 ^3^
Dyslipidemia	10 (6.1%)	9 (6.7%)	1 (3.4%)	>0.9 ^4^
Hyperparathyroidism	13 (8.0%)	9 (6.7%)	4 (14%)	0.3 ^4^
Hypothyroidism	7 (4.3%)	6 (4.5%)	1 (3.4%)	>0.9 ^4^
Primary Kidney Disease				
LN	7 (4.3%)	4 (3.0%)	3 (10%)	0.11 ^4^
DN	26 (16%)	23 (17%)	3 (10%)	0.6 ^4^
HN	33 (20%)	27 (20%)	6 (21%)	>0.9 ^3^
MPGN	1 (0.6%)	1 (0.7%)	0 (0%)	>0.9 ^4^
MN	7 (4.3%)	5 (3.7%)	2 (6.9%)	0.6 ^4^
FSGS	4 (2.5%)	3 (2.2%)	1 (3.4%)	0.5 ^4^
Cystinosis	1 (0.6%)	1 (0.7%)	0 (0%)	>0.9 ^4^

DM1: Type 1 diabetes mellitus; DM2: Type 2 diabetes mellitus; HTN: Hypertension; LN: Lupus nephritis; DN: Diabetic nephropathy; HN: Hypertensive nephroangiosclerosis; MPGN: Membranoproliferative glomerulonephritis; MN: Membranous glomerulonephritis; FSGS: Focal segmental glomerulosclerosis; UTI: Urinary tract infection. ^1^ Median (min, max); n (%); ^2^ Wilcoxon rank-sum test; ^3^ Pearson’s Chi-square test; ^4^ Fisher’s exact test.

**Table 2:** Pathological characteristics, and Outcomes in kidney transplant recipients according to UTI diagnosis.

Characteristic	UTI (−) n = 134 ^1^	UTI (+) n = 29 ^1^	*p*-Value
Baseline eGFR, mL/min/m^2^	60.0 (5.0–134.0)	35.0 (9.0–94.0)	0.002 ^2^
Donor type			0.5 ^3^
Deceased	65 (49%)	16 (55%)	
Living	69 (51%)	13 (45%)	
Ischemia time, hours	9.9 (1.0–28.0)	8.7 (3.0–26.0)	0.6 ^2^
PRA, %			0.6 ^4^
0–10	130 (97%)	28 (97%)	
11–50	2 (1.5%)	1 (3.4%)	
51–100	2 (1.5%)	0 (0%)	
HLA-A, # matches			>0.9 ^4^
1	23 (68%)	6 (75%)	
2	11 (32%)	2 (25%)	
HLA-B, # matches			>0.9 ^4^
1	19 (90%)	5 (100%)	
2	2 (9.5%)	0 (0%)	
HLA-DR, # matches			0.4 ^4^
1	28 (93%)	5 (83%)	
2	2 (6.7%)	1 (17%)	
Kidney Graft loss	11 (8.2%)	1 (3.4%)	0.5 ^4^

DM1: Type 1 diabetes mellitus; DM2: Type 2 diabetes mellitus; HTN: Hypertension; LN: Lupus nephritis; DN: Diabetic nephropathy; HN: Hypertensive nephroangiosclerosis; MPGN: Membranoproliferative glomerulonephritis; MN: Membranous glomerulonephritis; FSGS: Focal segmental glomerulosclerosis; UTI: Urinary tract infection. ^1^ Median (min, max); n (%); ^2^ Wilcoxon rank-sum test; ^3^ Pearson’s Chi-square test; ^4^ Fisher’s exact test.

**Table 3:** Evolution of estimated glomerular filtration rate in kidney transplant recipients according to UTI diagnosis.

Characteristic	eGFR_1_	eGFR_2_	eGFR_3_	eGFR_4_	*p*-Value ^1^
UTI (−) (n = 134)	60.0 (5.0–134.0)	61.5 (4.0–137.0)	60.0 (5.0–128.0)	60.9 (6.0–128.0)	0.59
UTI (+) (n = 29)	35.0 (9.0–94.0)	39.0 (7.0–94.0)	30.0 (6.0–94.0)	29.0 (5.0–101.0)	0.68
*p*-value ^2^	0.002	0.002	<0.001	<0.001	

eGFR: Estimated glomerular filtration rate; UTI: Urinary tract infection; ^1^ Friedman test; ^2^ Wilcoxon rank-sum test.

**Table 4:** Clinical characteristics and microbial profile in kidney transplant recipients with UTI, stratified by sex.

Characteristic	Overall n = 29 ^1^	Female n = 18 ^1^	Male n = 11 ^1^	*p*-Value
Hospitalization	3 (10%)	1 (5.6%)	2 (18%)	0.5 ^2^
Sample source				>0.9 ^2^
Urine	13 (45%)	8 (44%)	5 (45%)	
Bladder catheter	16 (55%)	10 (56%)	6 (55%)	
Type of infection				>0.9 ^2^
HAI	20 (69%)	12 (67%)	8 (73%)	
CAI	9 (31%)	6 (33%)	3 (27%)	
Gram				
Negative	29 (100%)	18 (100%)	11 (100%)	-
Microorganism				
*E. coli*	17 (59%)	10 (56%)	7 (64%)	0.7 ^2^
*Klebsiella* spp.	9 (31%)	7 (39%)	2 (18%)	0.4 ^2^
*E. cloacae*	2 (6.9%)	1 (5.6%)	1 (9.1%)	>0.9 ^2^
*P. aeruginosa*	1 (3.4%)	0 (0%)	1 (9.1%)	0.4 ^2^

CAI: Community-acquired infection; HAI: Healthcare-associated infection. ^1^ n (%); ^2^ Fisher’s exact test.

**Table 5:** Clinical characteristics and microbial profile in kidney transplant recipients with UTI.

Characteristic	Overall n = 29 ^1^	Female n = 18 ^1^	Male n = 11 ^1^	*p*-Value
Susceptibility profile				
Fully susceptible	7 (24%)	4 (22%)	3 (27%)	>0.9 ^2^
Amikacin	24 (83%)	15 (83%)	9 (82%)	>0.9 ^3^
Meropenem	21 (72%)	14 (78%)	7 (64%)	0.4 ^3^
Gentamicin	16 (55%)	11 (61%)	5 (45%)	0.4 ^2^
Piperacillin-tazobactam	15 (52%)	10 (56%)	5 (45%)	0.7 ^2^
Cefepime	12 (41%)	8 (44%)	4 (36%)	0.7 ^2^
Ceftazidime	11 (38%)	7 (39%)	4 (36%)	>0.9 ^2^
Ampicillin-sulbactam	11 (38%)	8 (44%)	3 (27%)	0.5 ^2^
Cefotaxime	10 (34%)	7 (39%)	3 (27%)	0.7 ^2^
Levofloxacin	8 (28%)	5 (28%)	3 (27%)	>0.9 ^2^
Ciprofloxacin	7 (24%)	5 (28%)	2 (18%)	0.6 ^2^
Resistance profile				
Nonsusceptible classes	8 (0, 14)	6 (0, 13)	10 (0, 14)	0.2 ^3^
ESBL	9 (31%)	6 (33%)	3 (27%)	>0.9 ^2^
AmpC	1 (3.4%)	0 (0%)	1 (9.1%)	0.4 ^2^
CR	8 (28%)	3 (17%)	5 (45%)	0.2 ^2^
MDR	19 (66%)	13 (72%)	6 (55%)	0.4 ^2^
XDR	3 (10%)	1 (5.6%)	2 (18%)	0.5 ^2^

ESBL: Extended-spectrum beta-lactamases; AmpC: AmpC-type beta-lactamases; CR: Carbapenem resistance; MDR: Multidrug-resistant; XDR: Extensively drug-resistant. ^1^ n (%); median (min, max); ^2^ Fisher’s exact test; ^3^ Pearson’s Chi-square test.

**Table 6:** Risk factors associated with graft loss in kidney transplant recipients. Cox regression analysis.

Characteristic	HR ^1^	95% CI ^1^	*p*-Value
Age, years	0.93	0.79–1.09	0.4
Male sex	0.65	0.13–3.28	0.6
Institutional referral	9.7	2.03–17.9	0.010
HTN	0.18	0.02–1.53	0.12
HN	2.83	0.49–16.2	0.2
DN	2.40	0.12–46.0	0.6
LN	0.75	0.05–10.8	0.8
Baseline eGFR, mL/min/m^2^	0.95	0.92–0.97	<0.001
Living donor	1.28	0.19–8.72	0.8
Ischemia time, hours	0.92	0.76–1.10	0.4
PRA 0–10%	10.1	0.44–234	0.15
UTI (+)	0.11	0.04–2.72	0.2

HR: Hazard ratio; HTN: Hypertension; HN: Hypertensive nephroangiosclerosis; DN: Diabetic nephropathy; LN: Lupus nephritis; eGFR: Estimated glomerular filtration rate; PRA: Panel reactive antibodies; UTI: Urinary tract infection; ^1^ HR = Hazard ratio; CI = Confidence interval.

## Data Availability

The data presented in this study are available within this article. The anonymized data underlying the results can also be made available upon reasonable written request to the authors, accompanied by a justification for its use.
